# MicroRNA-21 in cancer-associated fibroblasts supports lung adenocarcinoma progression

**DOI:** 10.1038/s41598-018-27128-3

**Published:** 2018-06-11

**Authors:** Akiko Kunita, Shigeki Morita, Tomoko U. Irisa, Akiteru Goto, Toshiro Niki, Daiya Takai, Jun Nakajima, Masashi Fukayama

**Affiliations:** 1 Department of Pathology, Graduate School of Medicine, University of Tokyo, Tokyo, Japan; 2 Department of Cellular and Organ Pathology, Akita University Graduate School of Medicine, Akita, Japan; 3 Department of Integrative Pathology, Jichi Medical University, Tochigi, Japan; 4 Department of Clinical Laboratory, University of Tokyo Hospital, Tokyo, Japan; 5 Department of Thoracic Surgery, University of Tokyo Hospital, Tokyo, Japan

## Abstract

Cancer-associated fibroblasts (CAFs) interact closely with cancer cells, supporting their growth and invasion. To investigate the role of microRNA-21 (miR-21) in lung adenocarcinoma, and especially in its CAF component, *in situ* hybridisation was applied to samples from 89 invasive lung adenocarcinoma cases. MiR-21 expression was observed in both cancer cells and CAFs. When the patients were stratified by expression, miR-21 levels in CAFs (n = 9), but not in cancer cells (n = 21), were inversely correlated with patient survival; patients with miR-21^high^ CAFs exhibited lower survival than those with miR-21^low^ CAFs. The underlying mechanism was investigated *in vitro*. Conditioned medium (CM) from A549 lung cancer cells increased miR-21 expression in MRC-5 and IMR-90 lung fibroblasts through the transforming growth factor-β pathway, and induced CAF-like morphology and migratory capacity. MiR-21 up-regulation in lung fibroblasts induced a novel CAF-secreted protein, calumenin, as well as known CAF markers (periostin, α-smooth muscle actin, and podoplanin). Moreover, CM from the lung fibroblasts increased A549 cell proliferation in a calumenin-dependent manner. Thus, miR-21 expression in lung fibroblasts may trigger fibroblast trans-differentiation into CAFs, supporting cancer progression. Therefore, CAF miR-21 represents a pivotal prognostic marker for this scar-forming cancer of the lungs.

## Introduction

Cancer-stromal interactions play critical roles in cancer cell growth, invasion, metastasis, angiogenesis, and chemoresistance^1–3^. Cancer-associated fibroblasts (CAFs), or activated fibroblasts, are essential constituents of the cancer stroma that contribute to cancer progression and promote cancer cell growth. Although their origin remains controversial, CAFs are thought to be mainly derived from resting fibroblasts^4^, which are activated to trans-differentiate into myofibroblasts/CAFs by paracrine signalling initiated by cancer cells. Transforming growth factor β (TGF-β) is the most potent activator of fibroblast trans-differentiation. In a previous study, we identified sub-epithelial myofibroblasts (SMFs) located within the alveolar septa and beneath the *in situ* proliferation of lung adenocarcinoma cells. We postulated that at least some of these SMFs transform into CAFs at the “central scar”^5^, the central region of lung adenocarcinoma, which contains myofibroblasts/CAFs. CAFs have only recently been investigated with respect to their molecular, genetic, and epigenetic abnormalities in various organs, including the lungs. Since CAF abnormalities may represent a useful indicator for cancer prognosis, their complete characterisation should be pursued.

MicroRNAs (miRNAs) are a class of small (20–25 nucleotides in length), non-coding RNA species that play important roles in tuning various cellular functions, through the post-transcriptional regulation of target mRNAs. The roles of miRNAs in the development and progression of cancer cells are currently being intensively investigated. Although CAFs represent an attractive target for the development of novel drugs, studies have mainly focused on cancer cell-derived miRNAs, while the roles of stromal-derived miRNAs remain to be elucidated. Bronisz *et al*. demonstrated that the down-regulation of miR-320 in mammary stromal fibroblasts reprograms the tumour microenvironment by activating the pro-oncogenic secretome^6^. Moreover, Mitra *et al*. identified a set of three miRNAs (miR-31, miR-214, and miR-155) that reprogram normal fibroblasts into CAFs in ovarian cancer^7^.

In this study, we focused on miR-21. MiR-21 is a representative oncogenic miRNA (oncomiR) that is up-regulated in various cancers, including lung adenocarcinoma^8–12^. At the cellular level, previous studies have mainly focused on miR-21 overexpression in cancer cells, which promotes cellular proliferation, evasion of apoptosis, epithelial-mesenchymal transition, and invasion. However, the role of miR-21 in CAFs has begun to attract attention. The prognostic significance of miR-21 expression in CAFs has been recognised in several cancers^8,13^. In a colorectal cancer study, miR-21 expression was increased in stromal cells as compared to normal tissues, and the ectopic expression of miR-21 drove the trans-differentiation of fibroblasts into myofibroblasts and increased invasion *in vitro*^13^.

We hypothesised that miR-21 over-expression in lung fibroblasts supports lung adenocarcinoma tumour progression by up-regulating CAF activity. To examine the validity of our hypothesis, we performed *in situ* hybridisation (ISH) to evaluate miR-21 expression in formalin-fixed paraffin-embedded (FFPE) tissue sections of lung adenocarcinoma. Then, we further investigated the role of miR-21 in the interaction between lung adenocarcinoma cancer cells and lung fibroblasts *in vitro*. Notably, miR-21 was highly expressed in both CAFs and lung adenocarcinoma cells, and its expression in CAFs was inversely correlated with patient survival. Our data suggest that miR-21 expression in lung fibroblasts induces CAF phenotypes and plays an important role in the progression of lung adenocarcinoma. Therefore, it is a potential novel molecular marker for lung adenocarcinoma prognosis.

## Results

### High miR-21 expression in CAFs is associated with poor lung adenocarcinoma prognosis

**Figure 1:**
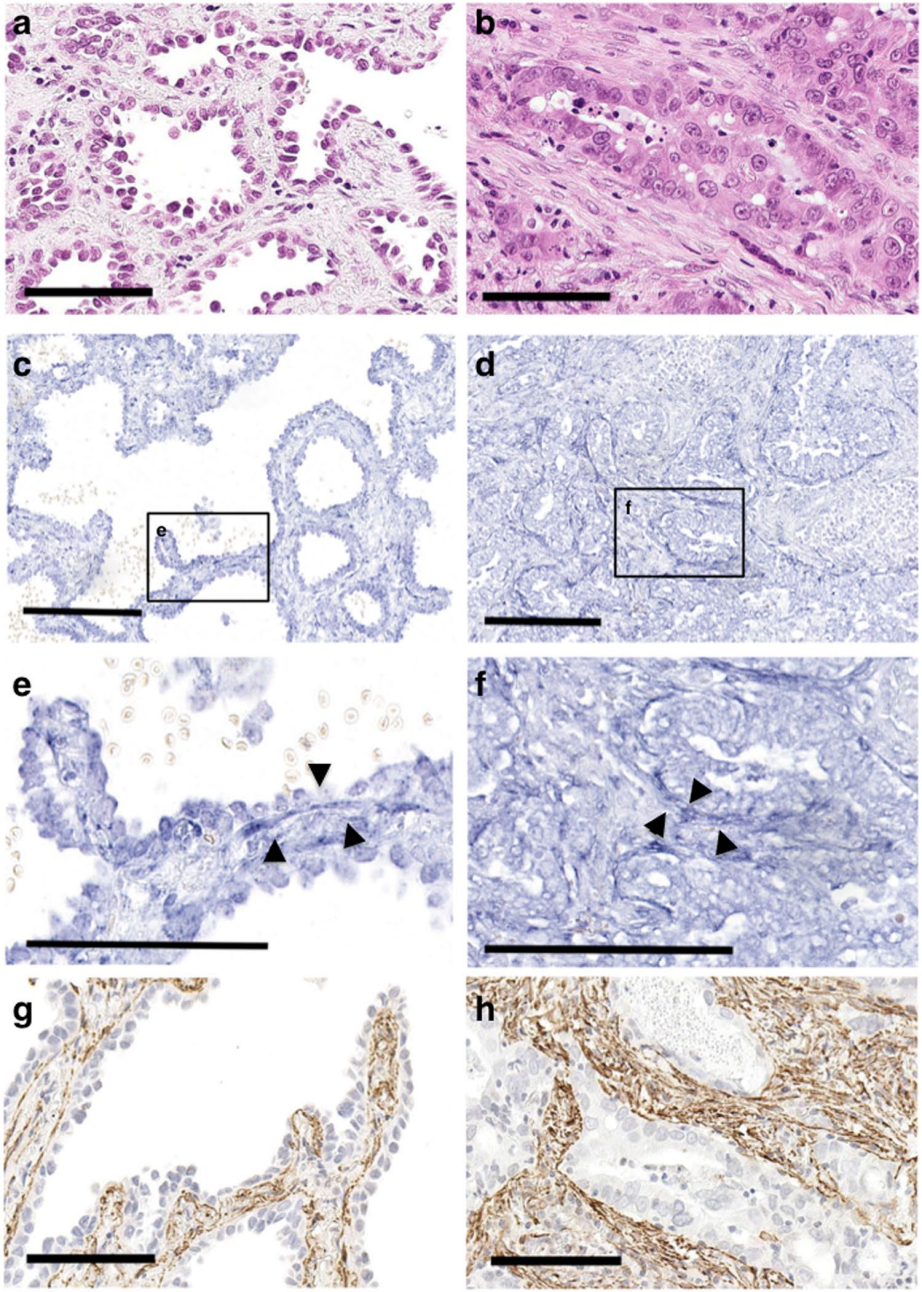
*In situ* hybridisation to detect miR-21 expression in lung adenocarcinoma cells. Non-invasive (lepidic pattern, **a**) and invasive (acinar pattern, **b**) areas in adenocarcinomas were evaluated using miR-21 *in situ* hybridisation (low power views: **c** and **d**, high power views: **e** and **f**) and α-SMA immunohistochemical staining (**g**,**h**). High miR-21 expression (3+) was observed in the cytoplasm of tumour cells in the non-invasive (**c**) and invasive (**d**) areas. Note the sub-epithelial fibroblasts in the non-invasive area showing low expression (1–2+) of miR-21 (**e**), and the stromal fibroblasts in contact with tumour cells in the invasive area showing high expression (3+) of miR-21 (**f**). MiR-21 expressing fibroblasts were positive for α-SMA in the non-invasive (**g**) and invasive areas (**h**). (**a**,**b)** Haematoxylin and eosin (H&E) staining. (**c**–**f)** MiR-21 *in situ* hybridisation. (**g**,**h)** α-SMA immunohistochemical staining (counterstained with haematoxylin). Scale bars, 100 μm.

ISH was performed to analyse miR-21 expression in both invasive and non-invasive areas of lung adenocarcinoma (Table 1, Fig. 1a,b) using FFPE sections of cancer tissues. The nuclear localisation of U6 small nuclear RNA (snRNA) was used as a positive control. A positive cytoplasmic signal for miR-21 (1–3+) was observed in both cancer (89% (79/89) in non-invasive and 92% (82/89) in invasive areas) and stromal cells (75% (67/89) in non-invasive and 81% (72/89) in invasive areas), and the signals varied in their distribution and abundance (Fig. 1c–f). In contrast, miR-21 signals were confined to scattered macrophages in non-neoplastic lung tissues (Supplementary Fig. S1). Notably, stromal fibroblasts that displayed high miR-21 expression overlapped with myofibroblasts positive for α-smooth muscle actin (α-SMA; Fig. 1g,h). This confirmed that the miR-21-expressing fibroblasts were SMFs in the non-invasive region, and CAFs in the invasive region. Additionally, cancer cells that exhibited high miR-21 expression were frequently in contact with miR-21-expressing CAFs in the invasive area (Fig. 1d). No signal was detected in collapsed and hyalinised fibrosis, which was devoid of cancer cells.

**Table 1:** MiR-21 expression in the non-invasive and invasive areas.

	miR-21 expression (tumour cells)		miR-21 expression (stroma)	
	Non-invasive	Invasive	Non-invasive	Invasive
0	10	7	22	17
1+	52	30	47	34
2+	19	31	15	29
3+	8	21	5	9
Low (0–2+)	81	68	84	80
High (3+)	8	21	5	9

**Figure 2:**
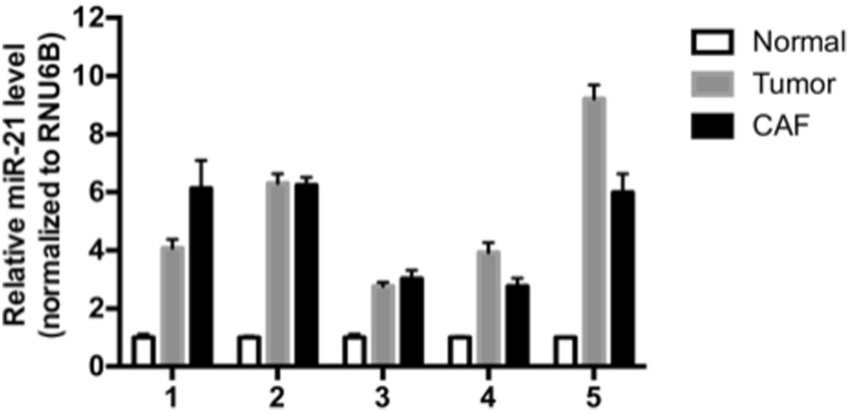
MiR-21 up-regulation in cancer cells and CAFs in lung adenocarcinoma. Cancer cells and stromal CAFs were extracted from formalin-fixed paraffin embedded sections by laser micro-dissection (five cases). The samples were subjected to TaqMan^®^ RT-qPCR analysis of miR-21. MiR-21 expression levels are presented as fold increases compared to the expression in samples from the adjacent non-cancerous area (mean ± SD, n = 3).

Positive and negative ISH signals were confirmed by quantitative reverse transcription-polymerase chain reaction (RT-qPCR). Cancer cells and stromal cells, which both showed 3+ positive signals, were separately extracted from the FFPE sections of five cases by laser micro-dissection (LMD) and were subjected to miR-21 analysis by RT-qPCR. MiR-21 expression was 5.26- and 4.83-fold higher in the lung adenocarcinoma tumour and stromal samples, respectively, when compared to adjacent, histologically normal tissue (*P* < 0.0005, Fig. 2).

**Figure 3:**
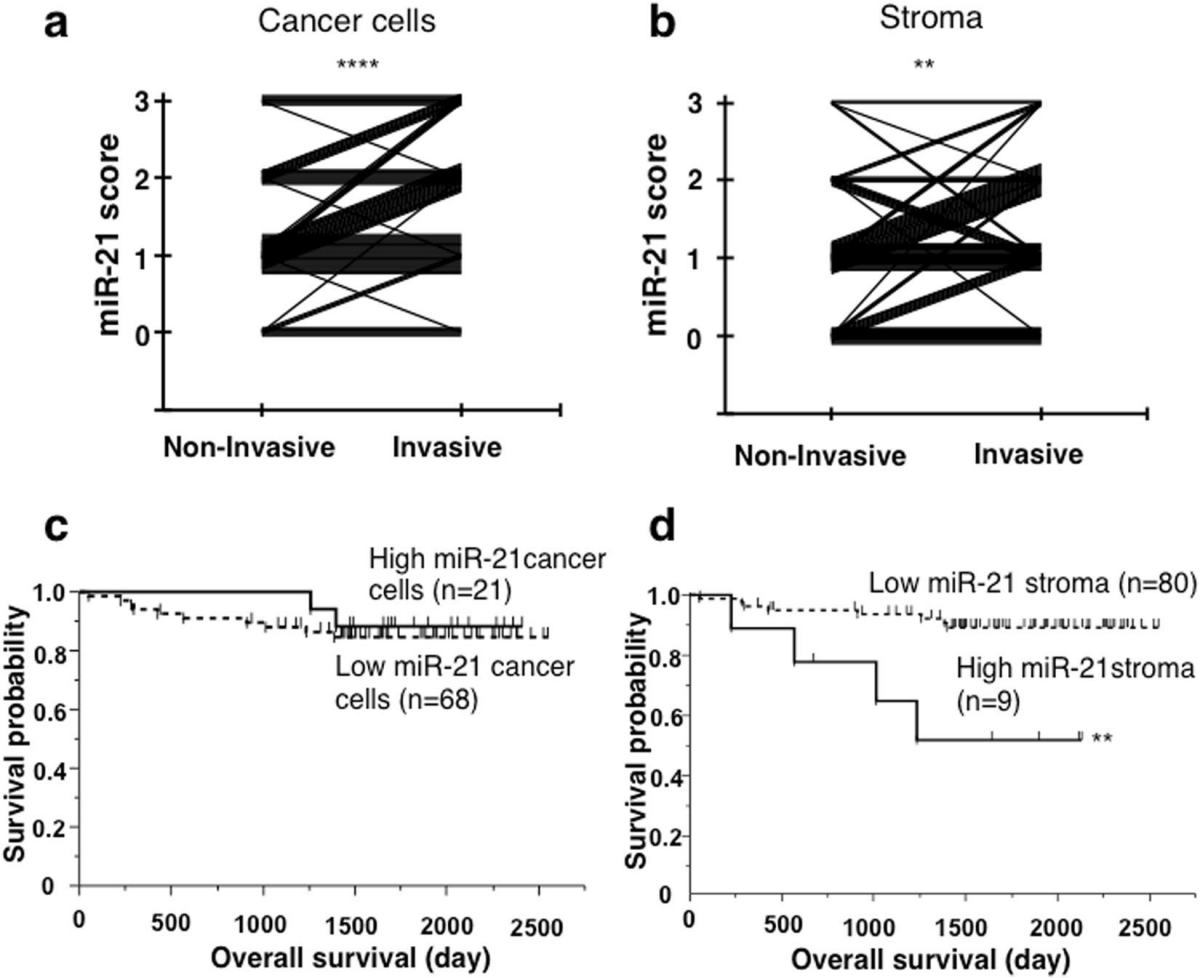
MiR-21 expression in the stroma of invasive areas in lung adenocarcinomas is associated with poor prognosis. (**a**,**b**) Scores for miR-21 expression in cancer cells (**a**) and CAFs (**b**) in non-invasive and invasive areas. Note the increase in scores in the invasive areas compared to those of the corresponding non-invasive areas (**a**: ****P < 0.0001, **b**: **P = 0.0041, Wilcoxon signed-rank test). (**c**) Overall survival of cancer cells expressing high or low levels of miR-21 in the invasive area. No significant differences were found (*P* = 0.5550). (**d**) Overall survival in stromal cells expressing high or low levels of miR-21. Stromal cells highly expressing miR-21 showed a significantly shorter survival (**P = 0.0021). The five-year overall survival rate was 51.9% in the high expression group, and 89.2% in the low expression group.

When miR-21 expression was graded, expression in cancer cells was higher in the invasive area than in the non-invasive area (Table 1 and Fig. 3a, *P* < 0.0001, Wilcoxon signed-rank test). Similarly, miR-21 expression was higher in the CAFs (stroma) than in the SMFs (Table 1 and Fig. 3b, *P* = 0.0041). The correlation between miR-21 expression scores in the non-invasive and invasive areas was statistically significant (Fig. 3a, Spearman’s rank correlation; ρ = 0.6691, *P* < 0.0001), and a similar correlation was observed for miR-21 expression in SMFs and CAFs (Fig. 3b, ρ = 0.3704, *P* = 0.0004). The correlation between miR-21 expression in cancer cells and CAFs was significant in the invasive areas (ρ = 0.2275, *P* = 0.0320), but not in the non-invasive areas (ρ = −0.0168, *P* = 0.8756).

No differences in clinico-pathological factors between lung adenocarcinoma cases with miR-21^high^ and miR-21^low^ cancer cells or between lung adenocarcinoma cases with miR-21^high^ and miR-21^low^ CAFs were observed (Table 2). Using Kaplan-Meier survival analysis, no difference in survival was detected between lung adenocarcinoma cases with miR-21^high^ and miR-21^low^ cancer cells (Fig. 3c, *P* = 0.5550). However, a significantly shorter overall survival was observed in patients with lung adenocarcinoma with miR-21^high^ CAFs compared to those with miR-21^low^ CAFs (Fig. 3d, *P* = 0.0021). For disease-free survival, no significant difference was observed (data not shown). Subsequent Cox proportional hazards multivariate regression analysis identified sex (hazard ratio (HR) = 15.93, *P* = 0.0002), the T factor (HR = 4.167, *P* = 0.0176), and expression of miR-21 in CAFs in the invasive area (HR = 5.494, *P* = 0.0158) as independent prognostic factors (Table 3).

**Table 2:** Clinico-pathological features of low and high miR-21 expression cases.

	miR-21 expression (tumour cells)		*P*	miR-21 expression (stroma)		*P*
	low (0–2+)	high (3+)		low (0–2+)	high (3+)	
Cases	68	21		80	9	
Age (mean ± SD)	65.8 ± 9.3	66.2 ± 10.9	0.8613	65.5 ± 9.9	68.9 ± 7.2	0.3408
Sex (M/F)	31/37	11/10	0.5857	37/43	5/4	0.5960
Smoking index (mean ± SD)	347.7 ± 565.3	294.2 ± 439.4	0.7047	335.6 ± 535.6	337.8 ± 592.7	0.7059
T (1/2/3/4)	41/23/2/2	17/1/3/0	0.8543	48/28/2/2	5/3/0/1	0.0894
N (0/1/2/3)	54/10/4/0	18/1/2/0	0.7207	64/10/6/ 0	8/1/0/0	0.0628
Stage (I/II/III/IV)	52/7/8/0	17/1/3/0	0.9769	62/8/10/0	7/1/1/0	0.0628
EGFR mutation (wt/mt/NA)	28/23/17	7/10/4	0.3268	31/29/20	4/4/1	0.9294

SD, standard deviation; M, male; F, female; EGFR, epidermal growth factor receptor; wt, wild type; mt, mutation; NA, not available.

**Table 3:** Univariate analysis and multivariate Cox regression analysis of various factors for prognosis of lung adenocarcinoma patients.

Variables	Hazard ratio (95%confidence interval)	Unfavourable/Favourable	*P*
**Univariate analysis**			
Age	1.163 (0.371–3.932)	≧65/<65	0.7950
Sex	14.67 (2.850–268.2)	M/F	0.0003*
Smoking Index	3.058 (0.873–11.96)	≧400/<400	0.0799
T	3.437 (1.081–12.88)	T2–4/T1	0.0362*
N	1.152 (0.303–7.498)	N1,2/N0	0.8525
Stage	2.759 (0.868–10.34)	≧IB/IA	0.0857
EGFR mutation	2.446 (0.679–11.35)	wt/mt	0.1751
miR-21 ISH(stroma)	5.580 (1.482–17.83)	high/low	0.0139*
miR-21 ISH(tumour cells)	1.573 (0.415–10.23)	high/low	0.5388
**Multivariate analysis**			
Sex	15.93 (3.042–293.1)	M/F	0.0002*
T	4.167 (1.280–16.01)	≧T2/T1	0.0176*
miR-21 ISH	5.494 (1.431–18.17)	High/low	0.0158*

EGFR, epidermal growth factor receptor; ISH, *in situ* hybridisation; M, male; F, female; wt, wild type; mt, mutation; *P < 0.05.

### A549 lung cancer cell culture medium increases miR-21 expression in lung fibroblasts

**Figure 4:**
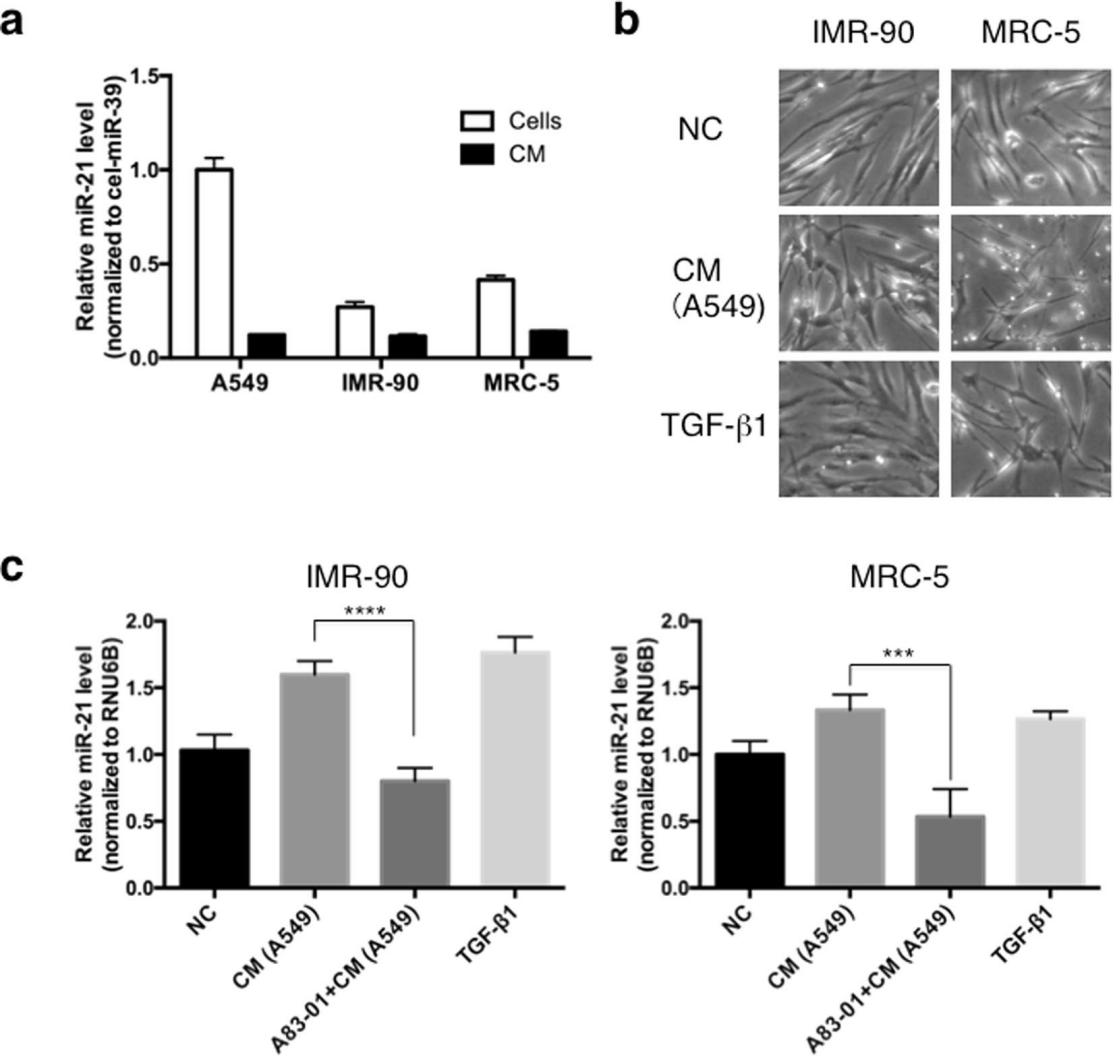
TGF-β1 induces myofibroblast differentiation and activates miR-21 expression in lung fibroblasts. (**a**) Comparative expression of miR-21 in A549 lung cancer cells, IMR-90 and MRC-5 lung fibroblast cells, and their conditioned media (CM, mean ± SD, n = 3). (**b**) Morphological changes following the stimulation of lung fibroblasts with TGF-β1 for 24 h. (**c**) MiR-21 expression in lung fibroblasts treated with CM from lung cancer A549 cells or with TGF-β1, with and without A83-01 (A83-01 + CM; mean ± SD, n = 3, ***P < 0.001, ****P < 0.0001).

To investigate the effects of miR-21 in the trans-differentiation of fibroblasts into CAFs, we used a fibroblast-to-CAF conversion model, where human normal lung IMR-90 or MRC-5 fibroblasts were cultured in the presence of TGF-β1 or conditioned medium (CM) from the A549 lung adenocarcinoma cell line. First, we determined the basal expression levels of miR-21 in these cells; miR-21 expression in A549 lung cancer cells was higher than in IMR-90 or MRC-5 lung fibroblasts, while miR-21 levels in the CM were comparable between A549, IMR-90, and MRC-5 cells (Fig. 4a). Morphological changes, such as swelling of the cytoplasm, were observed in MRC-5 and IMR-90 cells treated with TGF-β1 (Fig. 4b), which is the most potent factor driving fibroblast trans-differentiation into CAFs. Conversely, treatment with CM derived from A549 lung cancer cells had modest effects on IMR-90 cells, and induced thin protrusions in MRC-5 cells. Under the same experimental conditions, the expression of miR-21 in human lung fibroblasts increased 1.6- (IMR-90) and 1.3-fold (MRC-5) after 24 h in the presence of A549-derived CM, and increased 1.8- (IMR-90) and 1.3-fold (MRC-5) following treatment with TGF-β1, the positive control (Fig. 4c). The induction of fibroblast miR-21 expression by CM from A549 cells was abrogated by treatment with the TGF-β1 receptor inhibitor A83-01. These results suggested that the induction of lung fibroblast miR-21 expression by A549 lung cancer cell CM is modulated through the TGF-β1 pathway. The TGF-β1 concentration in the A549 cell CM was 45.2 ± 9.56 pg/mL (mean ± SD).

### MiR-21 induces CAF features and increases the migration of lung fibroblasts

**Figure 5:**
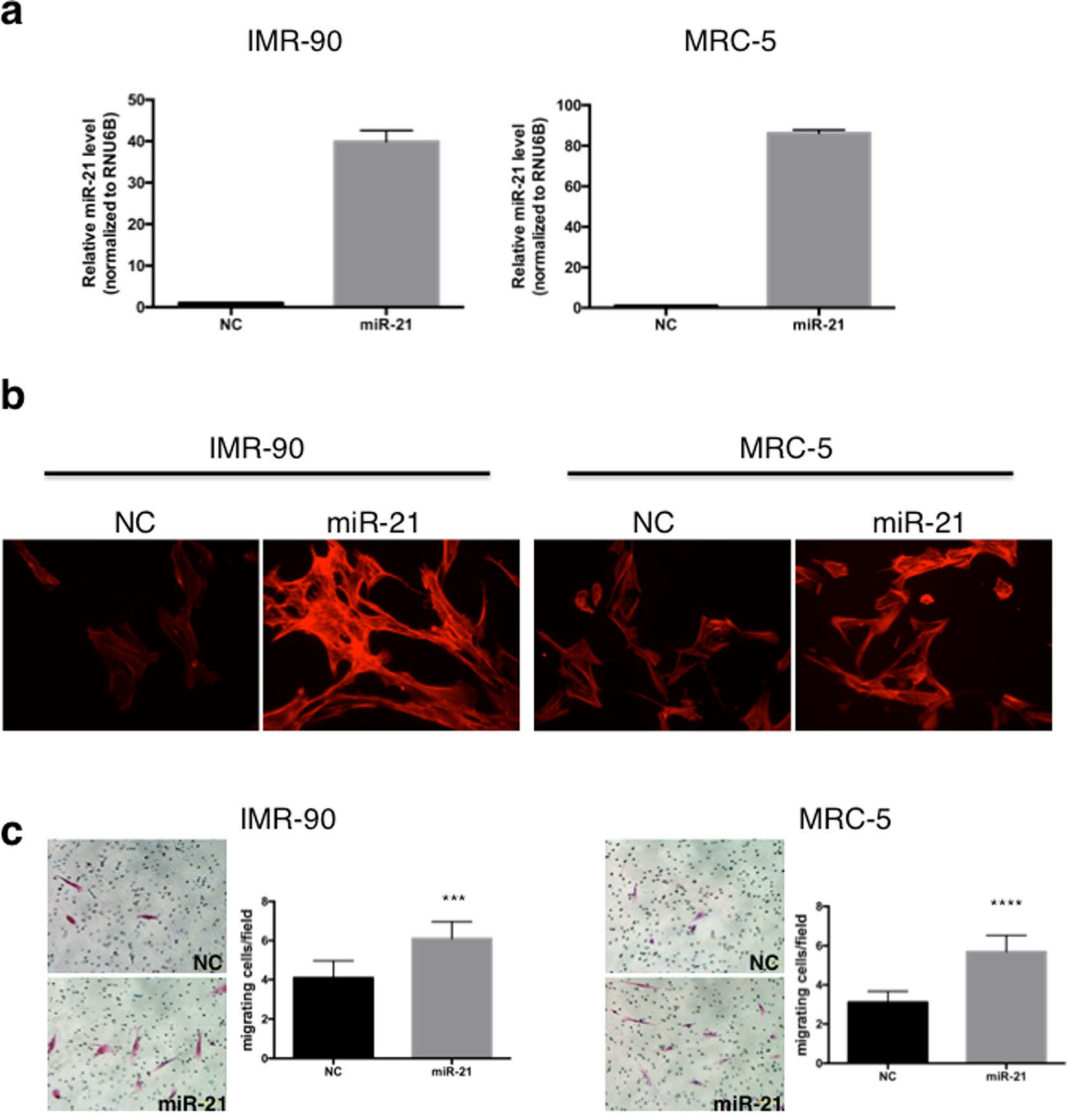
MiR-21 promotes stress fibre formation and the migration of lung fibroblasts. (**a**) MiR-21 overexpression by miR-21 mimic transfection into IMR-90 and MRC-5 cells was confirmed by TaqMan^®^ RT-qPCR (mean ± SD, n = 3). NC, negative control mimic. (**b**) Stress fibre formation in IMR-90 and MRC-5 cells expressing miR-21. Cells were stained for filamentous actin (Rhodamine-Phalloidin; red). Images were acquired at a magnification of 200×. (**c**) The migration of miR-21-overexpressing IMR-90 or MRC-5 cells was quantified by counting the number of H&E-stained cells remaining after the removal of non-migrated cells (mean ± SD, n = 10, ***P = 0.0002, ****P < 0.0001). Representative mages are shown on the left.

To investigate the effects of miR-21 on lung fibroblasts, miR-21 mimic (miR-21) or negative control mimic (NC) was transfected into IMR-90 or MRC-5 cells, and the cells were stained with phalloidin to visualize filamentous actin. Overexpression of miR-21 was confirmed by TaqMan^®^ RT-qPCR (Fig. 5a). MiR-21 expression was increased by 39.9- and 86.2-fold in IMR-90 and MRC5 cells, respectively, compared to the negative control. Increased levels of actin stress fibres, a known feature of CAFs, were observed in miR-21-expressing fibroblasts (Fig. 5b). Cell migration was also induced by miR-21 overexpression (Fig. 5c). Taken together, the results suggested that miR-21 induced a CAF-like phenotype in lung fibroblasts.

### Effects of CM derived from miR-21-transfected fibroblasts on A549 cells

**Figure 6:**
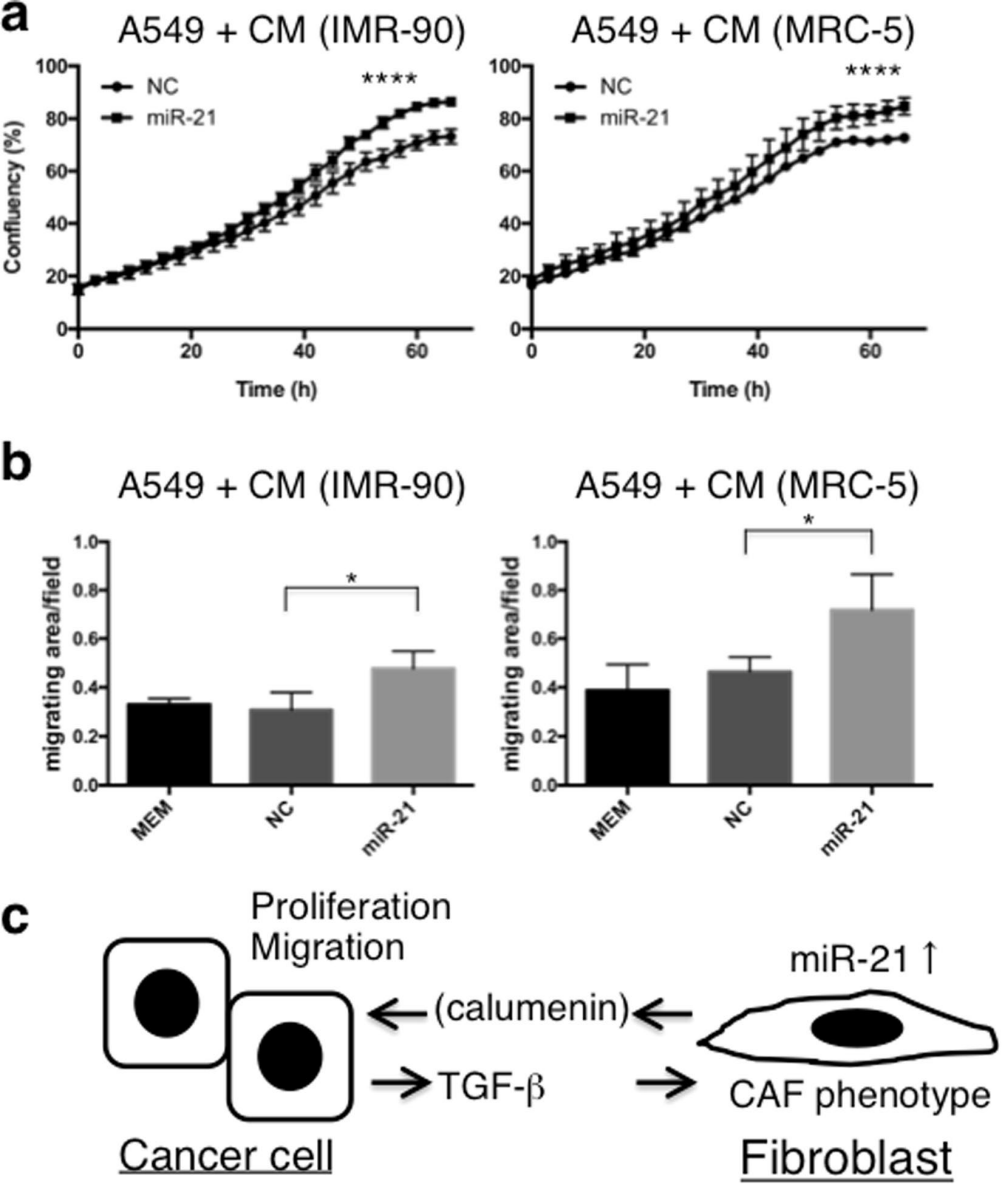
MiR-21-expressing lung fibroblasts affect the growth and migration of lung cancer cells through secretory molecules. (**a**) Cell proliferation assay for A549 cells following treatment with CM from miR-21-overexpressing IMR-90 and MRC5 fibroblasts (mean ± SD, n = 3, ****P < 0.0001). The proliferative capacity of lung cancer cells, cultured in CM from fibroblasts transfected with miR-21, was increased compared with the control group (NC). (**b**) A549 cell migration assay following treatment with CM from miR-21-overexpressing IMR-90 or MRC5 fibroblasts. Cell migration was analysed with the IncuCyte^®^ ZOOM live cell imager and expressed as the area occupied by A549 cells on the bottom surface, normalised to the initial top value (mean ± SD, n = 4). (**c**) Schema of the interactions between cancer cells and fibroblasts in lung adenocarcinoma.

Next, we evaluated the effects of CM derived from miR-21-transfected IMR-90 (IMR-90^miR-21^) or MRC-5 (MRC-5^miR-21^) cells on the proliferation and migration of A549 lung cancer cells. When A549 cells were cultured in CM derived from IMR-90^miR-21^ or MRC-5^miR-21^ cells, their proliferation and migration increased compared to the control group (Fig. 6a,b). Thus, these three experiments (Figs 4, 5 and 6) indicated that certain secretory products of CAFs enhance the growth and migration of cancer cells in lung adenocarcinoma. Such factors are inducible by TGF-β1 and depend on CAF miR-21 (Fig. 6c).

### Calumenin isinduced by TGF-β and miR-21 in lung fibroblasts

**Figure 7:**
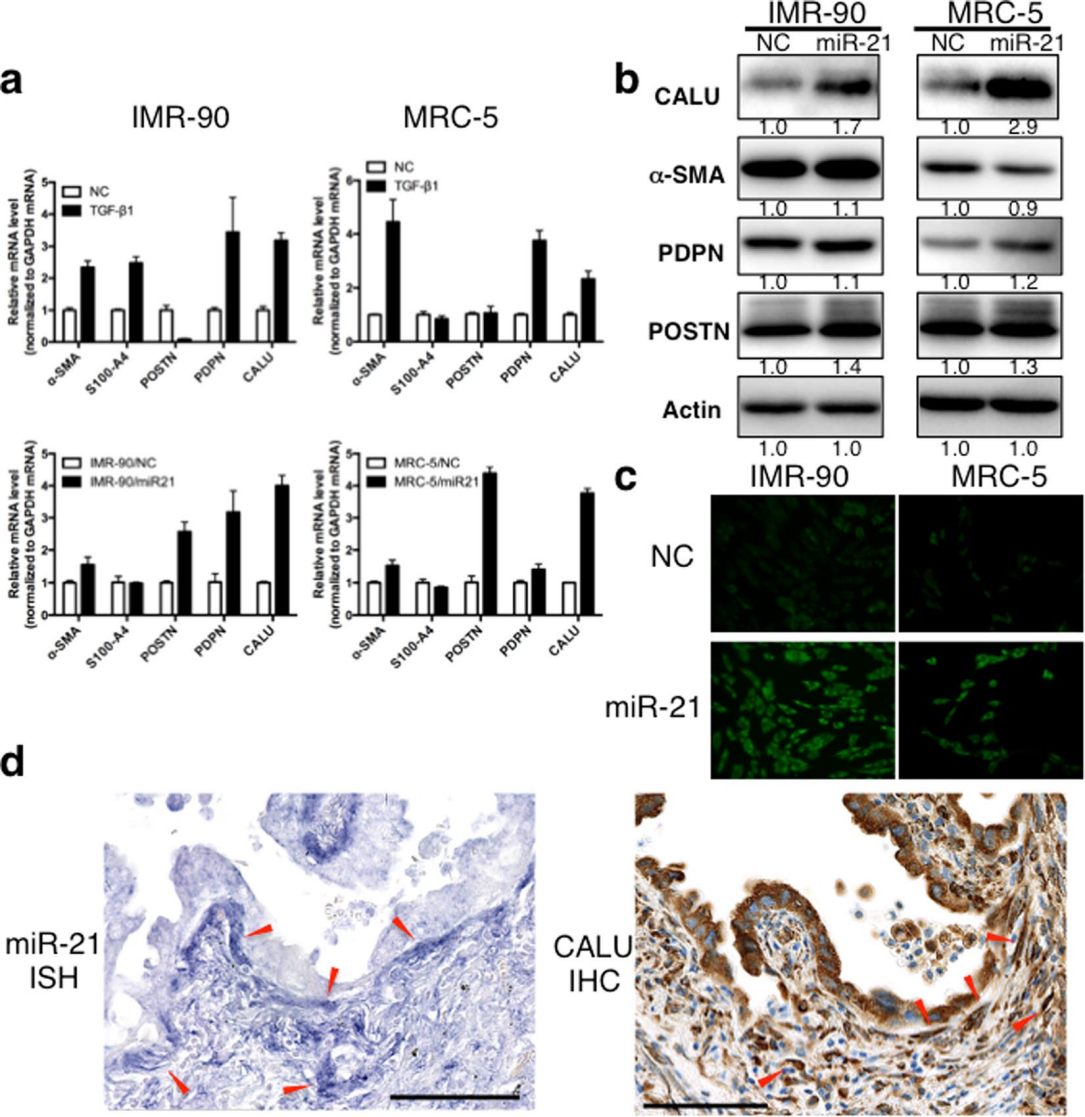
MiR-21 up-regulation in lung fibroblasts induces a novel CAF marker, calumenin. IMR-90 or MRC-5 lung fibroblasts transfected with miR-21 mimic (miR-21) or miRNA mimic negative control (NC) were collected for RT-qPCR, western blot, and immunofluorescence analyses. (**a**) IMR-90 or MRC-5 cells were treated with TGF-β1 for 24 h and the expression of CAF markers was evaluated by RT-qPCR (upper panels). Expression levels of CAF markers were examined by RT-qPCR in IMR-90 cells expressing the miRNA mimic negative control (IMR-90^NC^) or miR-21 mimic (IMR-90^miR-21^), and in MRC-5 cells expressing the miRNA mimic negative control (MRC-5^NC^) or miR-21 mimic (MRC-5^miR-21^; lower panels, mean ± SD, n = 3). (**b**) Western blot analysis of CAF markers in IMR-90^NC^ and IMR-90^miR-21^ cells (left) and in MRC-5^NC^ and MRC5^miR-21^ cells (right). Cropped images from the same blots are shown. Full-length blots are presented in Supplementary Fig. S2. Densitometry was performed and the signal intensities were normalized to Actin and expressed as fold change as compared to NC samples. (**c**) Immunofluorescent staining of miR-21-induced calumenin expression in IMR-90 and MRC-5 lung fibroblasts. Calumenin staining is shown in green. Images were acquired at a magnification of 200×. (**d**) Calumenin is expressed in cancer cells and in miR-21-expressing stromal cells in lung adenocarcinoma. Arrowheads indicate the miR-21-positive stromal area. MiR-21 ISH (left) and immunohistochemistry for calumenin (right) in lung adenocarcinomas from the same FFPE block are shown. Scale bars: 100 μm.

Next, we assessed the expression profiles of myofibroblastic markers. Recently, calumenin was identified as a secreted protein expressed in CAFs in colon cancer, and high stromal expression of calumenin was correlated with lymph node involvement and poor survival^14^. TGF-β1 treatment increased the expression of α-SMA, podoplanin (PDPN), and calumenin in both IMR-90 and MRC-5 cells (Fig. 7a, upper panel). Moreover, when the miR-21 mimic was transfected into IMR-90 or MRC-5 cells, a marked increase in calumenin mRNA was observed (Fig. 7a, lower panel). We further confirmed these results by measuring calumenin protein levels in both cell lines by western blotting (Fig. 7b) and immunofluorescence (Fig. 7c). MiR-21 induced calumenin protein expression in both IMR-90 (1.7-fold) and MRC-5 cells (2.9-fold). However, the other molecules (α-SMA, PDPN, and POSTN) were not profoundly induced by the expression of miR-21. Furthermore, the expression of calumenin in lung adenocarcinoma tissue samples was examined by immunohistochemistry. Calumenin was expressed in cancer cells and in miR-21-expressing stromal cells (Fig. 7d).

### Lung cancer-derived TGF-β induces calumenin up-regulation in lung fibroblasts, which in turn induces cancer cell proliferation

**Figure 8:**
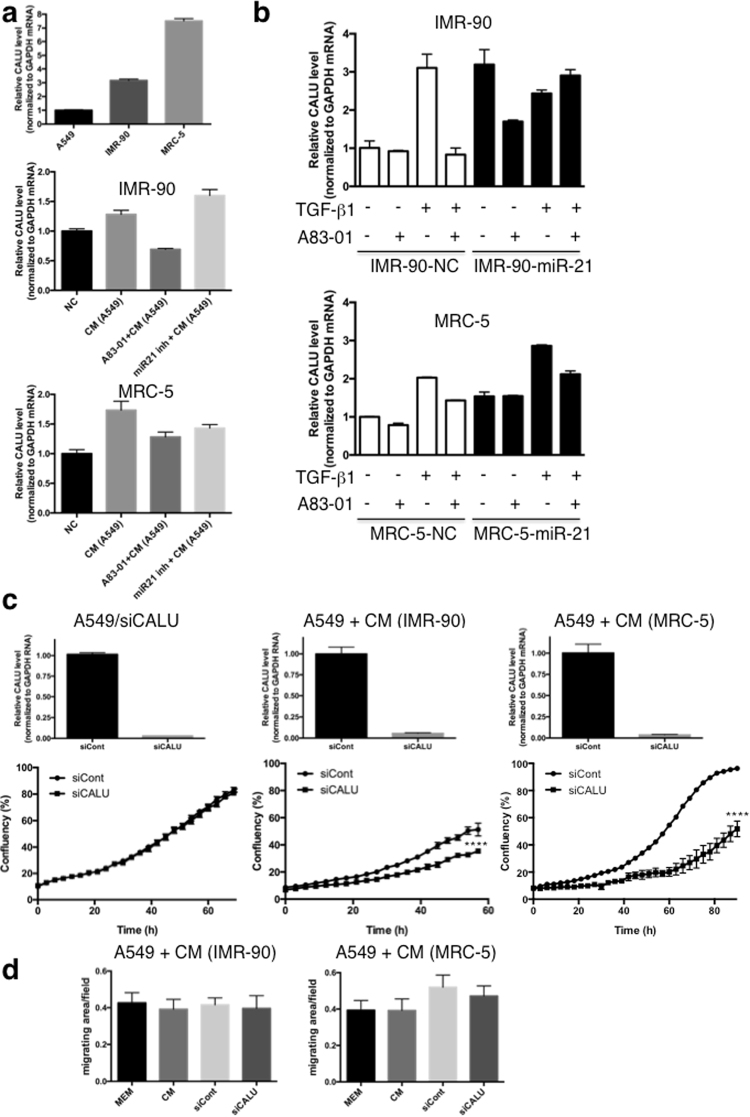
Calumenin up-regulation in lung fibroblasts: TGF-β1 induction, miR-21-mediated regulation, and effects on lung cancer cells. **(a**) Relative calumenin levels (normalised to glyceraldehyde 3-phosphate dehydrogenase (GAPDH)) in A549, IMR-90 or MRC-5 cells were evaluated by RT-qPCR (upper panel, mean ± SD, n = 2). Relative calumenin levels (normalised to GAPDH) in IMR-90 or MRC-5 cells treated with CM from lung cancer A549 cells without (CM (A549)) or with the A83-01 TGF-β receptor inhibitor (A83-01 + CM (A549)) or with miR21 inhibitor (miR21 inh + CM (A549)), compared to the normal condition (NC), were evaluated by RT-qPCR (middle and lower panels, mean ± SD, n = 3). (**b**) Relative calumenin levels (normalised to GAPDH) in miR-21-overexpressing IMR-90 or MRC-5 cells treated with TGF-β1 with and without A83-01 treatment were evaluated by RT-qPCR (mean ± SD, n = 3). (**c**) Cell proliferation assay of A549 cells following calumenin siRNA transfection (left, siCALU) or treatment with CM from IMR-90/calumenin siRNA (middle, siCALU) or MRC5/CALU siRNA (right, siCALU) fibroblasts (mean ± SD, n = 3, ****P < 0.0001). Calumenin knockdown efficiency was quantitatively evaluated by RT-qPCR (upper panels). The proliferative capacity of lung cancer cells, cultured in CM from fibroblasts transfected with calumenin siRNA, was decreased compared with the control group, whereas there was no effect on A549 cells transfected with calumenin siRNA (lower panels). (**d**) Migration assay of A549 cells using the IncuCyte^®^ ZOOM live cell imager following treatment with CM from IMR-90/calumenin siRNA (left, siCALU) or MRC5/calumenin siRNA (right, siCALU) fibroblasts. The data were expressed as the area occupied by A549 cells on the bottom surface, normalised to the initial top value (mean ± SD, n = 4).

We quantified calumenin expression in A549 lung cancer cells and IMR-90 or MRC-5 lung fibroblasts by RT-qPCR. In lung fibroblasts, the calumenin expression level was increased 3.2-fold (IMR-90) and 7.5-fold (MRC-5) as compared to A549 cells (Fig. 8a, upper panel). To investigate the induction of calumenin in lung fibroblasts, IMR-90 or MRC-5 cells were treated with CM from A549 lung cancer cells with or without the TGF-β1 receptor inhibitor A83-01 or the miR-21 inhibitors. In IMR-90 cells, the induction of calumenin by A549 cell CM was suppressed by A83-01 treatment but not by miR-21 inhibitors (Fig. 8a, middle panel). This suggested that the induction of **c**alumenin by CM from A549 cells was TGF-β1-, but not miR-21 -dependent. On the other hand, in MRC-5 cells, calumenin induction by CM from A549 cells was inhibited by both A83-01 and miR-21 inhibitor treatments (Fig. 8a, lower panel), indicating that calumenin induction was TGF-β1 - and miR-21-dependent.

To evaluate the effects of miR-21 on calumenin expression further, miR-21 mimic or NC was transfected into IMR-90 and MRC-5 cells (IMR-90^miR-21^, MRC-5^miR-21^, IMR-90^NC^, and MRC-5^NC^). TGF-β1 induced calumenin expression (3.1-fold in IMR-90 and 2.0-fold in MRC-5), which was inhibited by A83-01 in IMR-90^NC^ and MRC-5^NC^ cells (Fig. 8b). Calumenin expression was increased 3.2-fold in IMR-90^miR-21^ cells, and reduced to 1.7-fold by A83-01. Additional TGF-β1 did not increase calumenin expression, and A83-01 did not reduce calumenin expression in IMR-90^miR-21^ cells. In contrast, in MRC-5^miR-21^ cells, calumenin expression was increased, but not reduced by A83-01. On the other hand, in MRC-5^miR-21^ cells, TGF-β1 further induced the expression of calumenin, which was down-regulated by A83-01. These results suggested that exogenous TGF-β1 induces calumenin expression in lung fibroblasts in both miR-21-dependent and –independent manners. Knockdown of calumenin using small interfering (si)RNAs in A549 cells had no effect on cell growth (Fig. 8c, left panels). On the other hand, upon siRNA-mediated calumenin knockdown in both IMR-90 and MRC-5 cells (IMR-90^siCALU^ and MRC-5^siCALU^), the effects of CM on A549 cell proliferation were markedly suppressed (Fig. 8c, middle and right panels), while the migration of these cells was not affected (Fig. 8d). These results suggested that lung cancer-derived TGF-β1 increased the miR-21 levels in fibroblasts, which induced calumenin, and consequently promoted lung cancer cell proliferation.

## Discussion

Dysregulation of miR-21 has been reported in non-small cell lung cancers, including lung adenocarcinomas^15^. Although miR-21 is generally considered an oncomiR^12,16,17^, ISH indicated its expression by both cancer and stromal cells. This suggests that the roles of miR-21 in lung adenocarcinoma should be evaluated separately for each component. In fact, miR-21 expression was not completely correlated in the non-invasive and invasive regions, especially in stromal cells. MiR-21 expression in CAFs, but not in cancer cells, has been associated with a poor prognosis for lung adenocarcinoma. Since the prognostic value of CAFs has recently been recognised in various cancers, their further characterisation will be necessary for the stratification of patients with lung adenocarcinoma based on molecular, genetic, and epigenetic abnormalities.

In our previous study, we detected SMFs in the non-invasive region of lung adenocarcinoma, which are morphologically similar to CAFs in the invasive region. In this study, although the score was lower, miR-21 was expressed both in SMFs and in CAFs, and a significant correlation was observed for miR-21 expression between both types of myofibroblasts. This suggests that cancer cells can induce miR-21 expression in SMFs through undefined interactions between the two cell types. Under our experimental conditions, CM derived from A549 cells induced miR-21 expression in lung fibroblasts, and this increase was comparable to that observed following treatment with TGF-β1. Several theories have been postulated regarding the origin of CAFs, and one possibility is that resident fibroblasts or mesenchymal stem cells migrate from adjacent non-neoplastic lung tissues. Consistent herewith, miR-21- expressing SMFs, induced by TGF-β1 from cancer cells, may transform into CAFs by increasing miR-21 expression at the invasive front, where miR-21-expressing cancer cells augment miR-21 expression in CAFs.

An amplifying circuit has been proposed for miR-21 expression in myofibroblasts in pulmonary fibrosis. Liu *et al*. demonstrated that TGF-β1 induces miR-21 expression in lung fibroblasts, and that miR-21 in turn promotes TGF-β1-induced fibrogenic activation of pulmonary fibroblasts^18^. In the present study, we demonstrated that CM derived from A549 cells induced miR-21 expression in a TGF-β1-dependent manner and CAF-like characteristics, such as altered cellular morphology and migratory ability, in lung fibroblasts. Thus, miR-21 might be a key molecule inducing the common features of pulmonary fibrosis-myofibroblasts and lung adenocarcinoma CAFs. Further studies are needed to clarify how miR-21 is regulated by TGF-β1 in lung fibroblasts and CAFs. In renal fibrosis in rats, TGF-β-induced miR-21 up-regulation was mediated by Smad3 or sphingosine kinase/sphingosine-1-phosphate (SphK/S1P) signalling^19,20^. Thus, these molecules might be related to TGF-β-induced miR-21 up-regulation in lung fibroblasts.

CM collected from fibroblasts transfected with miR-21 increased both the proliferative and the migratory capacity of A549 cells. These *in vitro* data explain the aggressive behaviour of cancer cells *in vivo*, and support the use of CAF miR-21 expression as an independent prognostic marker for cancer prognosis. There are several mechanisms through which CAFs promote the proliferation of lung adenocarcinoma cancer cells, such as through the secretion of growth factors, metalloproteinases, secretory proteins, and even via miRNAs present in CAF exosomes (Fig. 6c). In the present study, we focused on calumenin, a previously reported stromal biomarker candidate with prognostic significance in colon cancer^21^. Although the precise mechanism of miR-21-mediated up-regulation of calumenin is unknown and might be cell-type dependent, the TGF-β-miR-21-calumenin pathway may be augmented by an autocrine mechanism of TGF-β-miR-21, as suggested by the results obtained in IMR-90 cells in the present study. Recently, calumenin was identified as a metastasis-related protein, which is highly expressed in metastasis-positive cases and facilitates the invasiveness of lung adenocarcinoma^14^. Moreover, calumenin is a multifunctional protein that reportedly inhibits cell migration^22^. Calumenin is a calcium-binding protein located in the endoplasmic reticulum (ER) that regulates ER functions, such as protein folding and sorting^23^. Calumenin has been reported to directly interact with SERCA2, which is the sole calcium-ATPase that transfers calcium into the ER, and its deregulation is tightly related to tumorigenesis^24^. Proteome studies in fibroblasts have shown that extracellular calumenin can influence actin polymerisation, inducing cytoskeletal rearrangements and cellular proliferation^25^. In this study, the proliferation of A549 lung adenocarcinoma cells was induced by CM from miR-21-expressing fibroblasts and suppressed by CM from calumenin knockdown fibroblasts. However, A549 cancer cell-derived calumenin had no effect on cancer cell growth. Thus, miR-21-expressing CAF-derived calumenin might function as an effector molecule in lung adenocarcinoma. Since increased cytosol calcium is able to promote cell proliferation via calcium-nuclear factor of active T cells (NFAT), we speculate that calumenin may modulate SERCA2 to promote proliferation in lung cancer cells. Further study is needed to clarify the functional mechanism of CAF-derived calumenin on cancer cell proliferation.

In conclusion, we have shown that miR-21 overexpression in CAFs has a prognostic impact on the survival of surgically treated patients with lung adenocarcinoma. Cancer cell-derived TGF-β1 induces the expression of miR-21 in lung adenocarcinoma fibroblasts, which may trans-differentiate into CAFs. MiR-21-expressing CAFs then promote the proliferation of cancer cells through the secretion of calumenin. Thus, miR-21 plays a key role in the progression of lung adenocarcinoma. Further studies will be necessary to elucidate the transition from SMFs to CAFs, which is important for understanding lung adenocarcinoma development.

## Methods

### Human tissue samples

Human tissue samples, including 89 lung adenocarcinoma samples, were obtained during pneumonectomy or lobectomy, without preoperative chemotherapy or radiotherapy, at the University of Tokyo Hospital between 2005 and 2010. Patients with lung adenocarcinoma were staged according to the tumour-node-metastasis system adopted by the American Joint Committee on Cancer and the International Union against Cancer. The cases consisted of 69 stage I (49 stage IA, 20 stage IB), 9 stage II (2 stage IIA, 7 stage IIB), and 11 stage III (8 stage IIIA, 3 stage IIIB) cases. This study was approved by the Ethics Committee of the Graduate School of Medicine of the University of Tokyo (No. 2381, G2211), and informed consent of was obtained from all the patients. All procedures were performed in accordance with the relevant guidelines and regulations.

### ISH for microRNA-21 and scoring

ISH was performed with the miRCURY LNA™ microRNA ISH Optimisation Kit for FFPE (Exiqon, Vedvæk, Denmark) based on the manufacturer’s instructions. After de-paraffinisation and proteinase K digestion, FFPE tissue sections were hybridised for 75 min at 52 °C with 5’-digoxigenin (DIG)-labelled miRCURY LNA™ microRNA detection probes for miR-21 (Exiqon), which were diluted to 40 nM in hybridisation buffer. U6 snRNA and scrambled probes were used as positive and negative controls, respectively. After incubation for 1 h at 23 °C with an alkaline phosphatase-conjugated anti-DIG Fab fragment, diluted 1:800 in blocking solution (Roche, Basel, Switzerland), the hybridised probes were detected by applying BCIP/NBT substrate solution (Roche) to the slides for 8 h. Tissues were counterstained with Nuclear Fast Red (Vector Laboratories, Burlingame, CA, USA).

The miR-21 staining results were classified into four grades according to Kadera *et al*.^26^: grade 0 (negative staining), grade 1+ (weakly positive), grade 2+ (moderately positive), and grade 3+ (strongly positive). Intra-alveolar macrophages served as internal positive controls (2+) for miR-21 staining, while the bronchial epithelium served as an internal negative control (0, Supplementary Fig. S1)^26^. Two pathologists independently evaluated fibroblasts in the invasive and non-invasive regions, and discrepancies were resolved in a joint session.

### LMD

Human lung adenocarcinoma FFPE specimens were sectioned to 20 μm thickness and placed onto membrane-mounted slides (Leica, Wetzlar, Germany). The slides were deparaffinised with xylene, rehydrated with ethanol, and stained with toluidine blue. LMD was performed with a pulsed ultraviolet laser on an LMD7000 system (Leica).

### Cell culture and CM

The A549 human lung adenocarcinoma cell line and the MRC-5 and IMR-90 human foetal lung fibroblast cell lines were obtained from the American Type Culture Collection (Rockville, MD, USA) or RIKEN BioResource Center (Tsukuba, Ibaraki, Japan) through the National Bio-Resource Project of the Ministry of Education, Culture, Sports, Science and Technology, Japan. A549 cells were grown in Dulbecco’s modified Eagle’s medium (DMEM) with l-Gln (Nacalai Tesque, Kyoto, Japan), and MRC-5 and IMR-90 cells were maintained in Minimum Essential Medium (MEM) with Earle’s Salts, l-Gln and Non-Essential Amino Acids (Nacalai Tesque), supplemented with 10% foetal bovine serum (FBS; CELLect, Lot No. 5766H; MP Biomedicals, Irvine, CA), 100 U/mL penicillin, and 100 μg/mL streptomycin (Nacalai Tesque), at 37 °C and 5% CO_2_ in a humid atmosphere.

CM from A549, IMR-90, and MRC-5 cells was produced by culturing cells in serum-free DMEM or MEM for 48 h. For TGF-β1 treatment, IMR-90 or MRC-5 cells were cultured in serum-free medium for one day, followed by the addition of TGF-β1 (R&D Systems, Minneapolis, MN, USA) at 5 ng/mL for 24 h. The concentration of TGF-β1 in A549 cell CM was analysed using the Human TGF-β1 Biotin-Free Immunoassay kit (AlphaLISA, Perkin Elmer, Inc., Waltham, MA, USA) according to the recommended protocol. In some cases, cells were treated with the TGF-β1 receptor inhibitor A83-01 (Wako Pure Chemical, Osaka, Japan) for 24 h before TGF-β1 administration.

### Transfection of miRNA mimics, miRNA inhibitors, and siRNAs

MiR-21 mimics (small, chemically modified double-stranded RNAs) were purchased from Ambion (Austin, TX, USA; mirVana™ miRNA Mimics). The mirVana™ miRNA mimic Negative Control #1 (Ambion) was used as a negative control. MiRNA inhibitors (Integrated DNA Technologies, IDT, Coralville, IA, USA) were used for inhibition of miR-21, along with the NC1 Negative Control (IDT). Cells were transfected with Lipofectamine^®^ RNAiMAX (Invitrogen, Life Technologies, Carlsbad, CA, USA) in the presence of miR-21 mimic (3 nM) or miR-21 inhibitor (10 nM). Overexpression or inhibition of miR-21 versus the negative control was confirmed by RT-qPCR using a TaqMan^®^ assay (Applied Biosystems, Foster City, CA, USA) 48 h after transfection.

For knockdown experiments, 5 nM of siGenome non-targeting siRNA Pool #1 or siGENOME SMARTpool siRNA containing a mixture of four siRNAs targeting calumenin (Dharmacon, GE Healthcare, Lafayette, CO, USA) were transfected using Lipofectamine^®^ RNAiMAX. The knockdown efficiency of calumenin siRNA was confirmed by RT-qPCR 48 h after transfection.

### RT-qPCR

Total RNA was isolated using ISOGEN II (Nippon Gene, Toyama, Japan) for cell culture samples or the Recover All Total Nucleic Acid Isolation Kit (Ambion, Foster City, CA, USA) for LMD samples, according to the manufacturers’ instructions. For the quantification of mature miR-21 in lung adenocarcinoma samples (A549, IMR-90, MRC-5 cells, their CMs, and the LMD samples), a TaqMan^®^ miRNA assay (Applied Biosystems) was used. Total RNA (10 ng) was reverse transcribed using miRNA-specific stem-loop primers. PCR was performed in triplicate using miR-21-specific primers and probes, using 2× Universal PCR Master Mix (Applied Biosystems) on an ABI 7500 qPCR instrument. Expression levels were analysed using the ΔΔCt method and were normalised to U6 snRNA levels. For comparative analysis of miR-21 in cells and CM, a mixture of 25 fmol of cel-miR-39 was spiked into all samples immediately after the addition of lysis buffer.

### Immunohistochemistry

Tissue samples were fixed in 4% neutral formalin and embedded in paraffin. Rabbit polyclonal anti-periostin (POSTN, ab14041, 1:1000; Abcam, Cambridge, UK), anti-α-SMA (1A4, 1:50; Dako, Glostrup, Denmark), anti-PDPN (LpMab-7^27^, 1:1000), and anti-calumenin (ab137019, 1:1000; Abcam) were used a primary antibodies. Immunohistochemistry was performed on 3 μm sections, as previously described^28^.

### Proliferation assay

To examine the growth of monolayer cultures, 5 × 10^4^ cells/well were seeded in 6-well plates, and cell confluence was monitored with the JuLI Stage automated cell imaging system and software (NanoEnTek, Seoul, Korea).

### Western blotting

Cell lysates were prepared in radio-immunoprecipitation buffer, separated by 10% sodium dodecyl sulphate-polyacrylamide gel electrophoresis, and transferred onto polyvinylidene difluoride membranes. Following blocking, the membranes were incubated overnight at 4 °C with anti-POSTN (1:500), anti-PDPN (1:1000), anti-calumenin (1:500), anti-α-SMA (1:500), or anti-β-actin (1:10000; Sigma) antibodies. The membranes were washed again and incubated for 1 h at RT with secondary antibodies. The antigen was then detected using the ImmunoStar Basic Chemiluminescence Reagent (WAKO Pure Chemical Industries Ltd, Osaka, Japan), according to the manufacturer’s instructions. Protein bands were captured by ChemiDoc Touch (Bio-Rad, Hercules, CA, USA). Signal intensities were quantified using ImageLab (6.0 version) software (Bio-Rad), normalized to their loading control β-actin, and expressed as fold change compared with controls.

### Immunofluorescence

Transiently transfected IMR cells (4 × 10^4^) were seeded in 4-well Falcon culture slides (Corning, NY, USA). After 72 h, the cells were fixed and permeabilised in 4% paraformaldehyde and 0.2% Triton™ X-100 in phosphate-buffered saline (PBS). After blocking for 20 min with 1% bovine serum albumin in PBS, incubation with the primary anti-calumenin antibody (1:500) was performed for 1 h at RT. After washing, the slides were incubated with a secondary Alexa Fluor^®^ 488 anti-rabbit antibody (1:250, Life Technologies) for 30 min at RT. A similar methodology was used to visualize the polymerised form of actin (F-actin) using Rhodamine-Phalloidin (Cytoskeleton Inc, Denver, CO, USA) staining. The slides were then mounted, counterstained with 4′,6-diamidino-2-phenylindole, visualised with a fluorescence microscope (Leica DM6000B; Leica Microsystems, Wetzlar, Germany), and processed using Leica LAS X software (Leica Microsystems).

### Migration assays

For IMR-90 or MRC-5 cell migration assays, 5 × 10^4^ cells were plated in serum-free medium in the upper chambers of Transwell^®^ inserts (8 μm pores; BD Biosciences, San Jose, CA, USA) in 6-well plates. The lower chambers were filled with complete medium and the cells were incubated for 24 h; after incubation, the cells in the upper chamber were removed using a cotton swab. The cells on the lower surface of the membrane were fixed with methanol, stained with haematoxylin and eosin (H&E), and counted.

A549 cell migration was quantified using the IncuCyte^®^ Chemotaxis Assay system (Essen Bioscience, MI, USA) following the manufacturer’s instructions. In brief, 2.5 × 10^3^ cells were plated in serum-free medium in the upper chambers (with 8 μm pores) of 96-well migration plates (IncuCyte^®^ ClearView, Essen Bioscience). The lower chamber was filled with CM from the lung fibroblast cell lines. Complete MEM including 10% FBS served as the assay control, and cells were incubated for 72 h. Whole-well images of the bottom of the plate membranes were captured using the IncuCyte^®^ ZOOM instrument.

### Statistical analysis

Statistical analyses were performed using JMP Pro (SAS Institute, Cary, NC, USA) or GraphPad Prism version 6 (GraphPad Software Inc., San Diego, CA, USA) software. The Wilcoxon signed-rank test, Mann-Whitney *U*-test, or one-way analysis of variance, followed by Tukey-Kramer multiple comparison testing, were performed, as appropriate. *P* values < 0.05 were considered statistically significant.

## Electronic supplementary material

Supplementary figures

## References

[1] Bhowmick NA (2004). TGF-beta signaling in fibroblasts modulates the oncogenic potential of adjacent epithelia. Science.

[2] Micke P, Ostman A (2004). Tumour-stroma interaction: cancer-associated fibroblasts as novel targets in anti-cancer therapy?. Lung Cancer.

[3] Mueller MM, Fusenig NE (2004). Friends or foes - bipolar effects of the tumour stroma in cancer. Nat. Rev. Cancer.

[4] Guo X, Oshima H, Kitmura T, Taketo MM, Oshima M (2008). Stromal fibroblasts activated by tumor cells promote angiogenesis in mouse gastric cancer. J. Biol. Chem..

[5] Matsubara D (2009). Subepithelial myofibroblast in lung adenocarcinoma: a histological indicator of excellent prognosis. Modern Pathol..

[6] Bronisz A (2012). Reprogramming of the tumour microenvironment by stromal PTEN-regulated miR-320. Nat. Cell Biol..

[7] Mitra AK (2012). MicroRNAs reprogram normal fibroblasts into cancer-associated fibroblasts in ovarian cancer. Cancer Discov..

[8] Kadera B (2013). MicroRNA-21 in pancreatic ductal adenocarcinoma tumor-associated fibroblasts promotes metastasis. J. Surg. Res..

[9] Nielsen BS (2011). High levels of microRNA-21 in the stroma of colorectal cancers predict short disease-free survival in stage II colon cancer patients. Clin. Exp. Metastasis.

[10] Nouraee N (2013). Expression, tissue distribution and function of miR-21 in esophageal squamous cell carcinoma. PLoS One.

[11] Rask L (2011). High expression of miR-21 in tumor stroma correlates with increased cancer cell proliferation in human breast cancer. APMIS.

[12] Seike M (2009). MiR-21 is an EGFR-regulated anti-apoptotic factor in lung cancer in never-smokers. Proc. Natl. Acad. Sci. USA.

[13] Bullock MD (2013). Pleiotropic actions of miR-21 highlight the critical role of deregulated stromal microRNAs during colorectal cancer progression. Cell Death Di.s.

[14] Nagano K (2015). Identification and evaluation of metastasis-related proteins, oxysterol binding protein-like 5 and calumenin, in lung tumors. Int. J. Oncol..

[15] Yanaihara N (2006). Unique microRNA molecular profiles in lung cancer diagnosis and prognosis. Cancer Cell.

[16] Zhang JG (2010). MicroRNA-21 (miR-21) represses tumor suppressor PTEN and promotes growth and invasion in non-small cell lung cancer (NSCLC). Clin. Chim. Acta.

[17] Liu XG (2012). High expression of serum miR-21 and tumor miR-200c associated with poor prognosis in patients with lung cancer. Med. Oncol..

[18] Liu G (2010). miR-21 mediates fibrogenic activation of pulmonary fibroblasts and lung fibrosis. J. Exp. Med..

[19] Liu X (2016). Transforming growth factor-beta-sphingosine kinase 1/S1P signaling upregulates microRNA-21 to promote fibrosis in renal tubular epithelial cells. Exp. Biol. Med..

[20] Zhong X, Chung AC, Chen HY, Meng XM, Lan HY (2011). Smad3-mediated upregulation of miR-21 promotes renal fibrosis. J. Am. Soc. Nephrol..

[21] Torres S (2013). Proteome profiling of cancer-associated fibroblasts identifies novel proinflammatory signatures and prognostic markers for colorectal cancer. Clin. Cancer Res..

[22] Zheng P, Wang Q, Teng J, Chen J (2015). Calumenin and fibulin-1 on tumor metastasis: Implications for pharmacology. Pharmacol Res..

[23] Honoré B (2009). The rapidly expanding CREC protein family: members, localization, function, and role in disease. Bioessays.

[24] Arbabian A (2011). Endoplasmic reticulum calcium pumps and cancer. BioFactors.

[25] Ostergaard M, Hansen GA, Vorum H, Honore B (2006). Proteomic profiling of fibroblasts reveals a modulating effect of extracellular calumenin on the organization of the actin cytoskeleton. Proteomics.

[26] Lu TX, Munitz A, Rothenberg ME (2009). MicroRNA-21 is up-regulated in allergic airway inflammation and regulates IL-12p35 expression. J. Immunol..

[27] Kato Y, Kaneko MK (2014). A cancer-specific monoclonal antibody recognizes the aberrantly glycosylated podoplanin. Sci. Rep..

[28] Kikuchi Y (2014). The niche component periostin is produced by cancer-associated fibroblasts, supporting growth of gastric cancer through ERK activation. Am. J. Pathol..

