# Supplementary material for: MicroRNA-21 in cancer-associated fibroblasts supports lung adenocarcinoma progression
Source: Sci Rep. 2018 Jun 11;8:8838. doi: 10.1038/s41598-018-27128-3 (PMC5995955; doi:10.1038/s41598-018-27128-3)
Supplement: Supplementary file 1 — Supplementary figures [file 41598_2018_27128_MOESM1_ESM.pdf]

MicroRNA-21 in cancer-associated fibroblasts supports progression of lung adenocarcinoma

Akiko Kunita, Shigeki Morita, Tomoko U. Irisa, Akiteru Goto, Toshiro Niki, Daiya Takai, Jun Nakajima, and Masashi Fukayama

Supplementary Information

Supplementary Figure S1
Supplementary Figure S2

Kunita et al.

Supplementary Figure S1. ***In situ*** **hybridization for microRNA-21 in the bronchopulmonary tissue.**
Low power view (**a** and **b**) of the non-neoplastic bronchus and its surrounding lung tissue, adjacent to the tumor (T). (**a**) Hematoxylin and eosin (H&E) staining. (**b**) *In situ* hybridization (ISH) for miR-21. Note the boxes indicating the paired panels of e and f, and g and h, respectively. ISH with snRNA U6 probe as a positive control (**c**) and ISH with scramble probes as a negative control (**d**). The bronchial epithelium (**e**, arrowheads) served as an internal negative control (0) (**f**, arrowheads). Intra-alveolar macrophages (**g**, arrows) served as internal positive controls for miR-21 staining (2+) (**h**, arrows). Bars: 200 μm (**a**-**d**); 100 μm, (**e**-**h**).

Supplementary Figure S2. Full-length blots

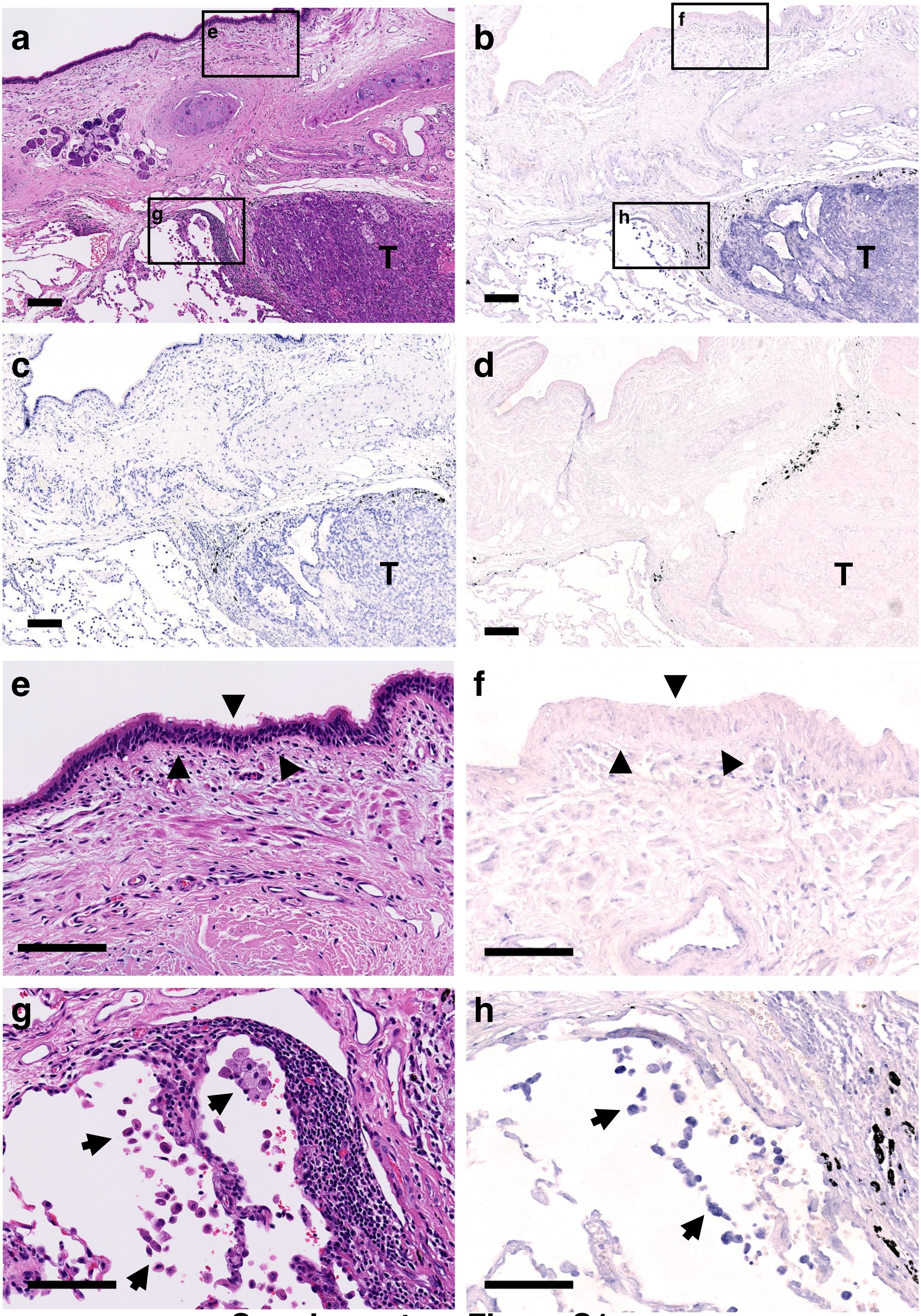

Supplementary Figure S1

CALU

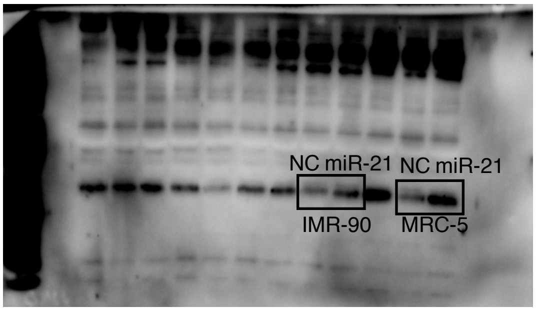

α-SMA

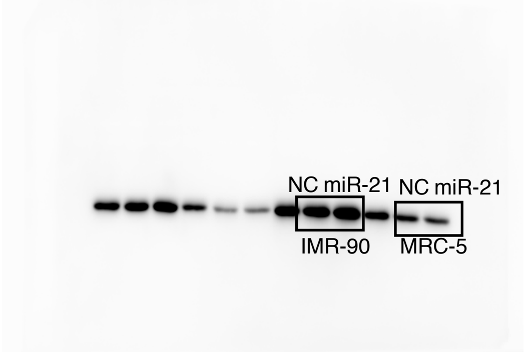

PDPN

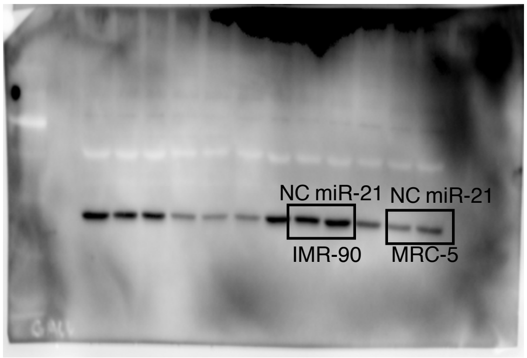

POSTN

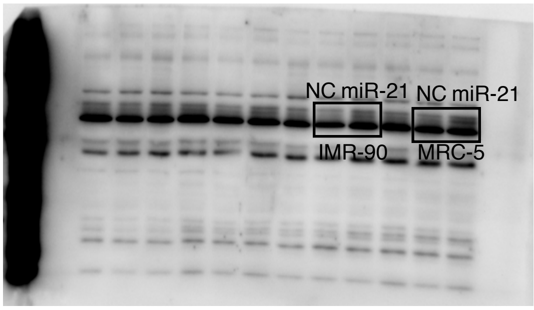

Actin

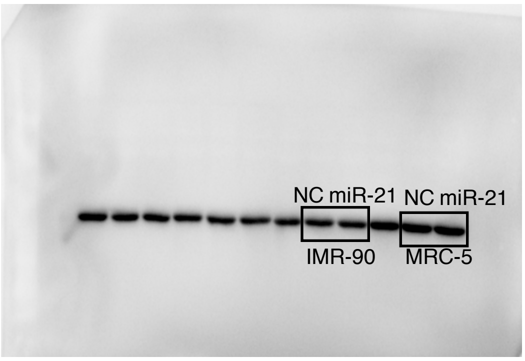

Supplementary Figure S2
